# Screening Patients With Cancer Admitted to Hanoi Medical University Hospital for Palliative Care Needs

**DOI:** 10.1200/GO.20.00102

**Published:** 2020-08-25

**Authors:** Quang V. Le, Huy L. Trinh, Kim Ngan T. Mai, Manh D. Pham, Paul A. Glare

**Affiliations:** ^1^ Department of Oncology and Palliative Care, Hanoi Medical University Hospital, Hanoi, Socialist Republic of Vietnam; ^2^ Hanoi Medical University, Hanoi, Socialist Republic of Vietnam; ^3^ Sydney Medical School, University of Sydney, New South Wales, Australia

## Abstract

**PURPOSE:**

To evaluate a screening tool for identifying which patients admitted to the oncology ward of a Vietnamese hospital should be referred to specialist palliative care (PC).

**METHODS:**

We performed a cross-sectional survey of consecutive patients hospitalized in the Department of Oncology and Palliative Care at Hanoi Medical University Hospital between June 2019 and September 2019. We translated a validated 11-item screening tool into Vietnamese and used a total score of ≥ 5 as a positive screen.

**RESULTS:**

One hundred participants were recruited. Forty-four patients (44%) screened positive. Of these, 37 (84%) had locally advanced or metastatic disease, 31 (70%) had uncontrolled symptoms, and 43 (98%) requested a PC consultation. A score ≥ 5 was significantly more common in patients with stage IV disease versus earlier stage, performance status of Eastern Cooperative Oncology Group (ECOG) 2 versus ECOG 0, and when life-limiting complications of cancer were present. Screening identified four patients overlooked by oncologists as needing referral, and 34% of patients requesting a referral had scores < 5.

**CONCLUSION:**

This screening tool provided oncologists with easy-to-use criteria for referring patients for PC. At the same time, it relieved the work load for under-resourced PC physicians by screening out requests with low-level need. This tool should be part of routine assessment on admission in all oncology units in Vietnam.

## INTRODUCTION

Palliative care (PC) is defined by the WHO as “an approach that improves the quality of life of patients and their families facing the problem associated with life-threatening illness, through the prevention and relief of suffering by means of early identification and impeccable assessment and treatment of pain and other problems, physical, psychosocial and spiritual.”^1^^(p94)^ PC is relevant in all life-limiting illnesses, but this is particularly the case in patients with cancer: a diagnosis of cancer can have a detrimental impact on both physical health and mental health of patients. In Vietnam, the estimated number of new cancer cases in 2018 was 164,671 (0.17% of the population of approximately 96 million), and the number of deaths was 114,871 (0.12% of the population),^2^ presenting a large burden of PC need nationally.

**CONTEXT**

**Key Objective**
To demonstrate the extent of the need for specialist palliative care in patients admitted to the oncology ward of a university medical center in Hanoi, using a validated screening tool for this purpose that was translated into Vietnamese.
**Knowledge Generated**
A total of 44% of 100 consecutive admissions screened positive for palliative care (score ≥ 5 out of 14). In this setting, it was most useful for screening out patients requesting a palliative care referral who did not really need it.
**Relevance**
This useful tool was quick and easy to use in the hands of oncologists who were familiar with the patients being screened and their families. The tool appears to have international transferability, as it has now been validated in the United States, Europe, and Asia.

In the past, PC was withheld until the final stages of cancer.^3^ Nowadays, the benefits of early referral to PC are recognized.^4^ Providing PC alongside life-prolonging tumor-directed treatment contributes to better oncology care for patients and families, in terms of better symptom management, quality of life, and satisfaction with care and less psychological distress; some studies even suggest survival benefit.^5^ The National Comprehensive Cancer Network (NCCN) Clinical Practice Guidelines in Oncology recommend screening all patients for PC needs and to call a PC consultation when any referral criteria are met.^6^ Unfortunately, workforce shortages, lack of support from management, and constrained PC program resources often present significant barriers to operationalizing these recommendations.^7^

PC is a relatively new concept in Vietnam. Although the first inpatient and outpatient services opened at the National Cancer Hospital (NCH) in Hanoi in 2001, there followed a long period in which skilled PC physicians were lacking.^8^ A 4-month specialist training program in palliative medicine was held in Hanoi in 2008 and in Ho Chi Minh City from 2011 to 2014, and graduates of these programs have been employed at a few hospitals. Currently, the three main centers of PC in Vietnam are the NCH in Hanoi, Hanoi Oncology Hospital, and Ho Chi Minh City Oncology Hospital. Most oncology teams in Vietnam lack an in-house specialist PC program, including our unit at Hanoi Medical University Hospital (HMUH), and oncologists are responsible for the providing basic PC and the identification, prioritization, and referral of patients who need specialist PC services.

However, a recent survey found that most physicians in Vietnam need more training in PC.^9^ The usual method of selecting patients for PC consultation is the oncologist’s clinical judgment of need. At HMUH, there are typically 300-400 admissions to the oncology ward annually, and we refer patients with specialist-level need to the NCH because of the absence of a PC specialist on site. A method for standardizing the process of identifying which patients to refer would be useful. Moreover, at HMUH, there is a high level of patient demand for PC because the name of the department is “Oncology and Palliative Care,” and many patients and families misunderstand it to be a type of anticancer treatment.

The aim of this study was to evaluate the feasibility and clinical usefulness of the Vietnamese translation of a validated PC screening tool,^10,11^ so that we may improve selection of patients for referral to our limited specialist PC resources. We hypothesized that the Vietnamese translation of the tool would be easy to incorporate into routine patient care and would be clinically useful to busy oncology house staff to systematically identify which patients would obtain the greatest benefit from specialist PC referral.

## METHODS

### Study Design

We undertook a cross-sectional survey of consecutive patients hospitalized in the Department of Oncology and Palliative Care at HMUH over a 4-month period in 2019. The survey commenced in June and continued until September, at which time 100 patients had been surveyed.

Of note, there is no institutional review board at HMUH or Hanoi Medical University. Therefore, the research was approved and supported by the Head of the Department of Oncology and Palliative Care, HMUH. Patients gave written consent to participate.

### Patients

Patients were recruited if they satisfied the following inclusion criteria: (1) adult (≥ 18 years old), (2) having first admission during the study period, (3) able to understand and cooperate with the study protocol, and (4) willing to participate and provide written informed consent. The exclusion criteria were: (1) multiple primary tumors, and (2) cancers of unknown primary.

### Measures: Screening Tool

**FIG 1:**
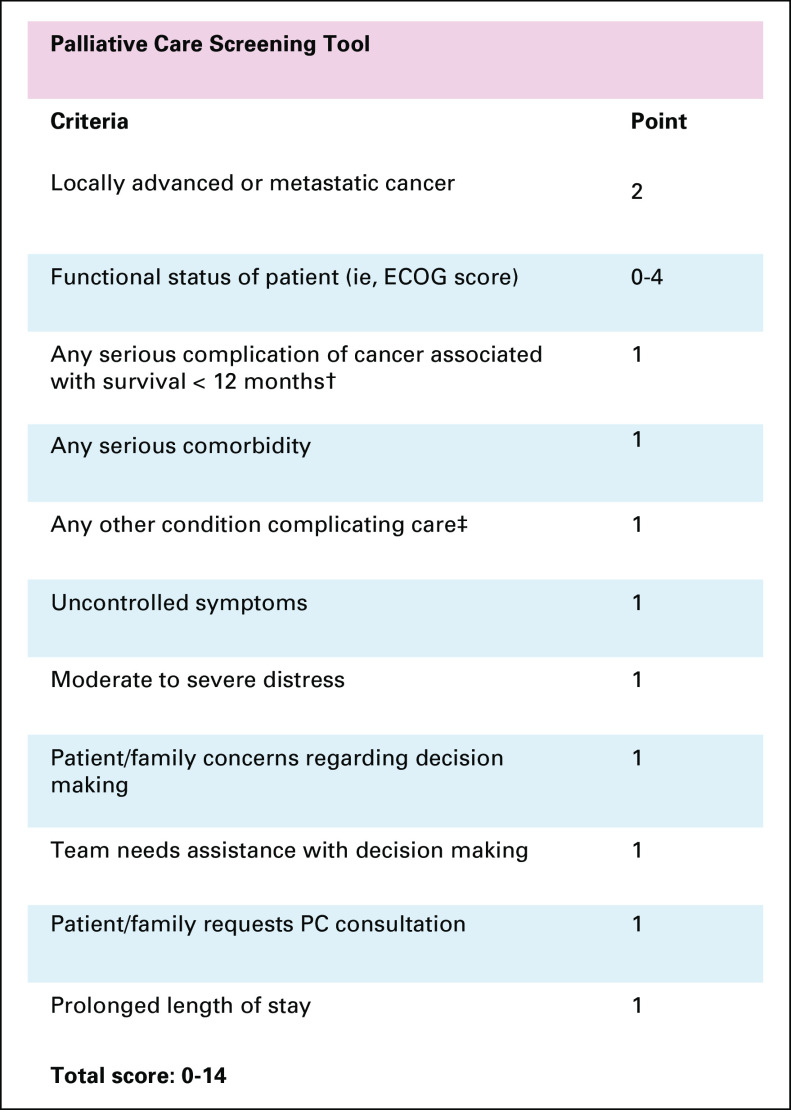
English version of the screening tool. (†) Examples given in National Comprehensive Cancer Network (NCCN) guideline include: Eastern Cooperative Oncology Group (ECOG) of 3 or Karnofsky performance score of 50, hypercalcemia, brain or CSF metastasis, delirium, superior vena cava syndrome, spinal cord compression, cachexia, malignant effusions, bilirubin ≥ 2.5 mg/dL, and creatinine ≥ 3 mg/dL. (‡) NCCN guideline does not specify these conditions. Palliative Care Center of the Bluegrass (Lexington, KY) suggests: liver disease, moderate or end-stage renal disease, moderate or advanced cardiac disease, moderate or advanced chronic obstructive pulmonary disease, stroke with loss of 50% of function, other life-limiting illnesses, or other conditions complicating care. PC, palliative care.

We used an 11-item, PC screening tool (Fig 1), which has been shown to be valid and feasible to use in routine inpatient oncology practice in the United States and when translated into German.^11,12^ We translated the tool into Vietnamese for convenience (Data Supplement).

### Data Collection Procedure

Eligible patients were screened with the tool 3 to 7 days after admission, to allow time for the results of any clinically relevant tests to be received. The data were collected by two medical oncologists who were familiar with the patients’ cases. They collected the data according to the following process:

- Item 1, extent of disease: The American Joint Committee on Cancer 8th TNM staging system stage was used.
- Item 2, performance status (PS): The Eastern Cooperative Oncology Group (ECOG) score was used to measure the PS.^13^
- Items 3-5, presence of life-limiting complications of cancer—prognosis < 12 months (eg, brain metastases), serious medical comorbidities (eg, congestive heart failure), or other conditions complicating care (eg, dementia, pressure sores).
- Information from items 1-5 were obtained from the medical record.
- Items 6-9, PC needs of patient or family: Uncontrolled symptoms, moderate to severe psychological distress, concerns regarding decision making, desire for a PC consultation: these were assessed by the clinicians and typically took approximately 5 minutes of discussion with the patient and/or their family to elicit them all.
- Item 10, assistance needed by treating team with clinical decision making.
- Item 11, assistance needed with discharge planning when there was a prolonged length of stay, defined as > 14 days at HMUH: This item was scored subsequently, at the time of discharge.

### Screening Process

After collecting these data, the total score for each individual patient was calculated (range, 0-14). We categorized patients into two groups on the basis of the total score: the first group comprised patients with a score of < 5 and the second group comprised patients with a score of ≥ 5. Five was chosen as the cut point on the basis of previous testing of the tool.^11,12^ In these studies, a score of ≥ 5 was highly predictive for meeting one or more of the National Comprehensive Cancer Network’s (NCCN) criteria for specialist PC referral.^6^ To determine the usefulness of the screening process, we sought to determine its yield, which we defined as the number of patients who screened positive who were not already referred to the specialist PC service.

### Statistical Analysis

Descriptive statistics were used to summarize the data. Comparative statistics were used to compare patients by clinical subgroups and screening scores above and below the cut point. Categorical variables were express as numbers (%) and compared by χ^2^ test or Fisher’s exact test between two groups. There were no continuous variables in our study. A two-sided α of < .05 was considered statistically significant. Statistical analyses were done using the IBM SPSS software (Armonk, NY), version 20.0.

## RESULTS

The demographic and clinical data of the 100 patients are summarized in Table 1. They included 51 males and 49 females, with a mean age of 60 years (range, 36-79 years). More than half of the patients had GI malignancies (colon 22%, stomach 19%, and rectum 13%). Locally advanced or metastatic disease was present in 52%. Although all had a PS of 0-2, 65 had cancer complications associated with a prognosis of < 12 months. In addition, 35 patients had serious medical comorbidities and 25 patients had other complex care needs.

**TABLE 1:**
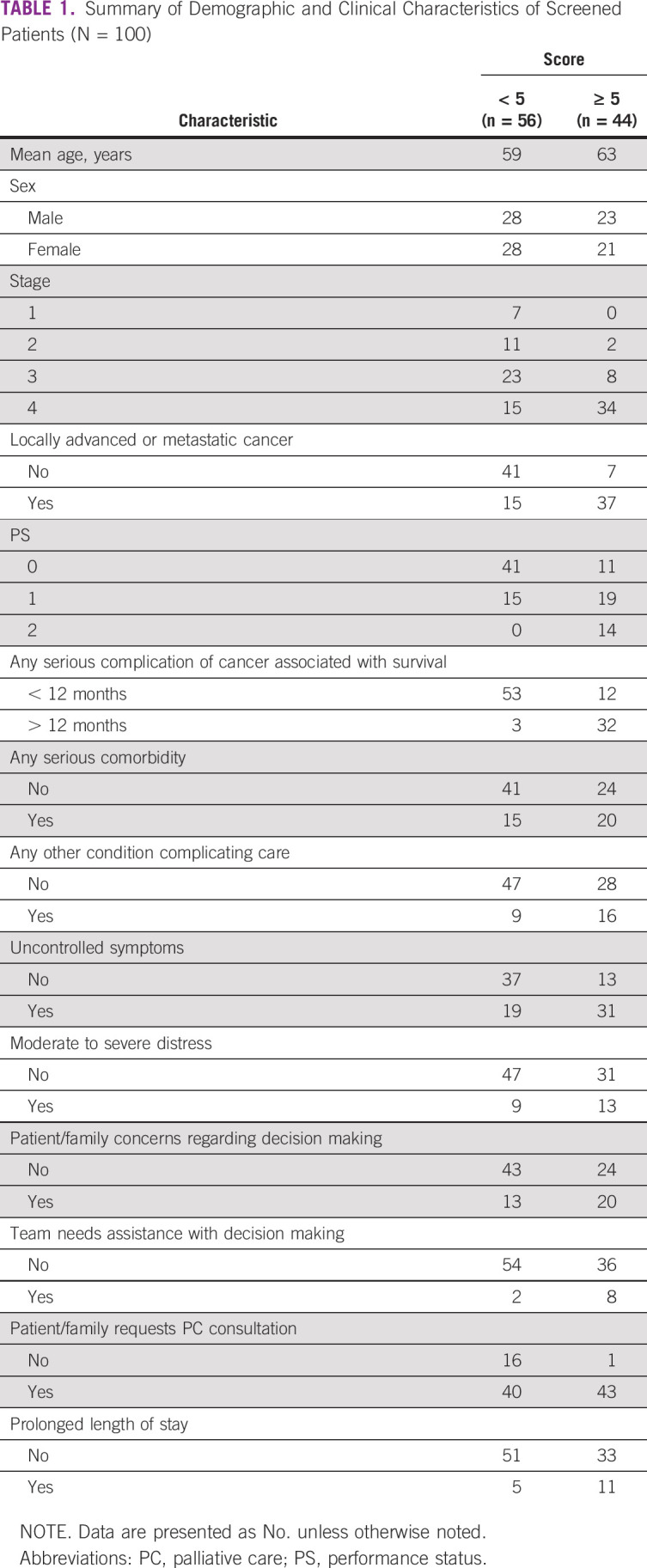
Summary of Demographic and Clinical Characteristics of Screened Patients (N = 100)

Table 1 also summarizes the PC needs of the patients and their families. Exactly half had poorly controlled physical symptoms, and 22 were suffering from moderate to severe distress. Approximately two out of three patients and/or families had concerns about clinical decision making, but in only 10% of cases did the treating oncology team believe they needed assistance from a PC specialist. Sixteen patients had been in the hospital for > 2 weeks.

### Differences Between Patients With Scores Above or Below the Cut Point

**FIG 2:**
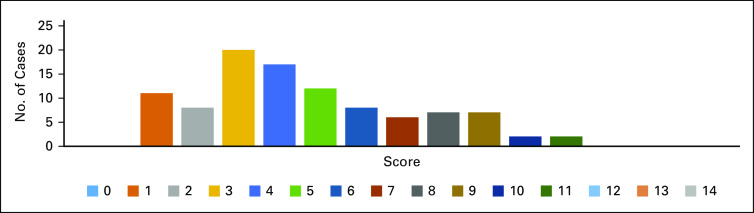
Distribution of screening scores.

The distribution of the scores is shown in Figure 2. Forty-four patients screened positive, with a total score of ≥ 5. Of them, 84% had locally advanced or metastatic disease, 70% had uncontrolled symptoms, and 98% requested a PC consultation. Additional characteristics of each group can be seen in Table 1.

### Subgroup Analysis According to Individual Factors: Tumor Stage, Performance Status, Primary Cancer Site, Cancer Complications, and Symptom Burden

#### Stage.

As shown in Table 2, nearly half of the patients had stage IV disease. Significantly more patients with stage IV disease had a score of ≥ 5 compared with those with stage I-III disease (69% *v* 20%; *P* < .01).

**TABLE 2:**
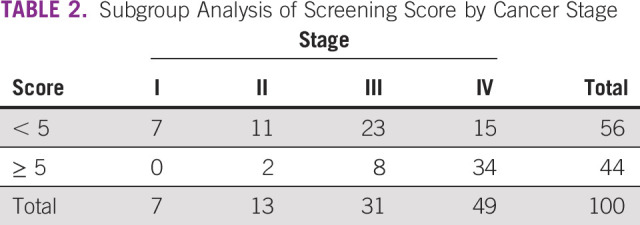
Subgroup Analysis of Screening Score by Cancer Stage

#### PS.

**FIG 3:**
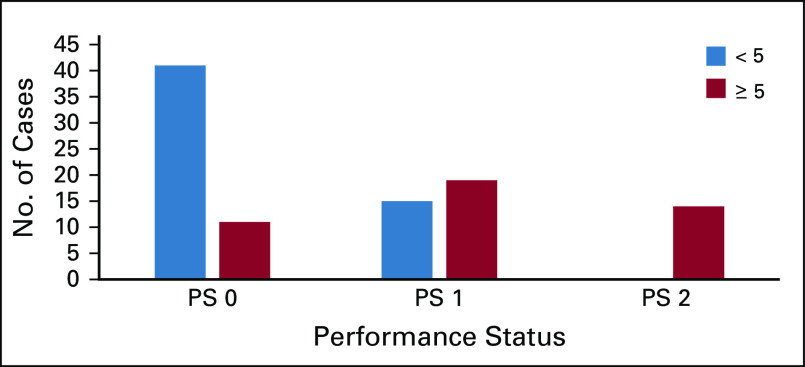
Subgroup analysis of score by performance status (PS).

Patients with PS score of ECOG 2 all scored ≥ 5, and most patients with PS score of ECOG 0 had total scores of < 5 (Fig 3). As a result, there was a statistically significant difference in the percentage of patients screening positive in the PS = 0 and PS = 2 groups (21% *v* 100%; *P* < .001).

#### Site of primary tumor.

**FIG 4:**
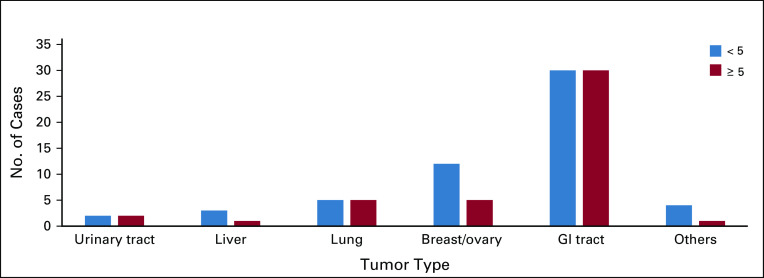
Subgroup analysis of score by primary tumor.

This was categorized into six groups (Fig 4). Our study comprises 60 patients having tumors initially located in the GI tract. Half of them had scores of ≥ 5. In the breast-ovary group, 12 patients had a score of < 5, compared with only five patients with a score of ≥ 5. Despite these trends, there was no significant difference in the percentage of patients having scores of ≥ 5 across the six groups. (*P* = .772).

#### Serious complications of cancer.

Cancer complications typically associated with survival of < 12 months were identified in approximately one-third (35%) of all participants. They were significantly more likely to have a score of ≥ 5 than those without life-limiting complications (91% *v* 18%; *P* < .001). As a result of undertaking the survey, 83 patients/families requested a PC consultation, but only 43 of them had a score of ≥ 5. On the other hand, only one of the 17 patients/families not requesting a PC consultation had a score of ≥ 5 (*P* = .001).

#### Uncontrolled symptoms.

Half of the patients had uncontrolled symptoms. Significantly less of them had score of < 5 than those in the controlled symptoms group (38% v 74%; *P* = .001).

#### Number of referrals.

The tool identified four new patients with a score of ≥ 5 whom their oncologist had not thought of referring. On the other hand, the tool also identified 22 (34%) of the 65 patients whom the oncologists intended to refer who had a score of < 5.

## DISCUSSION

Early integration of PC with oncology treatment is known to improve patient outcomes,^4^ but questions remain about the optimal timing and nature of PC integration.^14^ Needs-based integration may better capture patients who would benefit from referral than relying on disease-related factors but may lead to excessive referrals and strain the already limited PC workforce. Consequently, a blended, trigger-based approach that uses certain disease-based and prognosis-based triggers with screening of unmet needs has been recommended.^14^ The tool presented here fits such a construct.

In this study, we evaluated the Vietnamese translation of an existing English-language tool in a series of consecutive patients and we found it easy to use. Ultimately, 44% of all patients screened positive when the published score of ≥ 5 was used. This percentage was lower than previous evaluations of the tool in the United States and Germany.^11,12^ In the United States, the tool was developed and tested in patients hospitalized under the GI Oncology Service—also the most common tumor primary site in our survey—at a large US comprehensive cancer center (CCC). The GI Oncology Service was selected because they were perceived to have a high level of unmet PC need. Of them, 73% screened positive, with 64% of them meeting the NCCN’s criteria for referral to specialist PC criteria.^6,12^ The tool was shown to have good construct and criterion validity in that setting.^10^ The tool was subsequently translated into German for testing on inpatients at a CCC in the city of Erlangen.^11^ The Erlangen study reported the results for 208 patients prescreened with the first two items on the tool (advanced cancer and a poor prognosis) from a total population of 455 patients. Although 81% of these selected patients screened positive, the rate fell to 37%—similar to our results at HMUH—when the total 455 admissions were included. The German translation of the tool had good validity and good reliability as well. The scores were independent of age, sex, and primary diagnosis, but patients who had already been in contact with specialist PC had significantly higher screening scores than patients who had not yet been. The authors concluded that proxy assessment of specialist PC needs by oncologists is feasible and that the tool presents a valid instrument to trigger a specialist PC consultation.^11^

Several other approaches have been taken to address the challenge of identifying which oncology patients would benefit from referral to specialist PC. These have included triggers based on disease stage,^15,16^ hospitalization,^17^ and patient self-report of distress.^18^ However, the blended approach taken by the tool we evaluated here, combining disease factors and patient needs, is deemed to be the best approach,^14^ and our results support this view. For example, most patients with stage IV disease did have scores ≥ 5, but 30% did not. Meanwhile, 20% of patients with earlier-stage disease screened positive, and as they still have a chance to receive curative treatments and have longer overall survival, they have the most to gain from integrating PC with their oncologic treatment. Similar findings were observed when PS score, estimated overall survival, or poor symptom control were tested as single triggers for referral.

The clinical usefulness of any screening tool is measured by finding new, unrecognized cases. In this study, the tool only identified four patients whom the oncologists had not intended to refer: all had early-stage disease but a poor PS and serious medical comorbidities. Although the number was reassuringly small, it is important to identify such cases, as those individuals would be expected to obtain the most benefit of early integration of PC with their cancer treatment. Typically, 10%-15% of patients at HMUH ask for a PC referral. Although this is similar to the proportion in the Erlangen study,^8^ it does not reflect a similar level of awareness of PC in the community. Rather, patients and families at HMUH see the term “Palliative Care” in the name of our department and they think it is a modality of anticancer therapy. To this point, 83% of patients/families in our study requested PC consultation when we came to that item on the tool. Therefore, at HMUH it is helpful to have a tool that can identify patients and families requesting a PC consultation when they are unlikely to need it. Not only would such referrals be a poor use of the scare specialist PC resources in patients with early-stage disease, a good PS, and few other problems, but also they are burdensome for the patient, because the PC consultation requires transfer to the NCH, some 15 minutes away across town.

There are several limitations to the findings of this study. First, it was a small study in one unit in Hanoi with a historical interest in PC. The results may not be generalizable to other units in Vietnam. In particular, the prevalence of PC needs may be higher in other cancer units who have sicker patients (we had no patients with PS scores of 3-4).

Second, the data were captured at one point in time, close to admission, and may fail to take into account that PC needs may change over time (eg, if disease turns out to be more advanced than was known on admission or if new problems arise).

Third, we undertook screening for PC need but did not attempt to determine how many of the positive cases had basic PC needs that could be met by an oncologist with some training and given adequate resources^12^ and how many in fact required referral to a PC specialist. Finally, the patients were known to the oncologists performing the survey. Therefore, the opportunity cost in terms of staff time for performing the screening would be higher if it is performed by staff to whom the patients are less well known.

Having shown the tool is translatable into Vietnamese, it could be translated into other Asian languages and tested. It should be evaluated for validity and reliability in this setting. It should be tested against specialist PC referral criteria, such as those promulgated by the NCCN or others,^6,12^ to see how many screen-positive cases need referring and how many can be managed by the oncology team. It should be tested in other settings (eg, ambulatory patients)^19^ and in other Vietnamese hospitals that have more seriously ill patients.

In conclusion, this 11-item tool provided the oncologists at HMUH with an efficient method for identifying patients for referral to PC. It also helped reduce the workload of PC specialists who are overburdened by multiple referrals with low-level need, allowing them more time to provide patient-centered care to patients who needed it. On the basis of our experience using the tool, we recommend that PC screening should be part of routine assessment on admission, and all oncology units in Vietnam should adopt screening as soon as possible.
